# Editorial: New insights into autoinflammatory diseases: from bench to bedside

**DOI:** 10.3389/fmed.2023.1253150

**Published:** 2023-09-14

**Authors:** Wafa Ammouri

**Affiliations:** Department of Internal Medicine, Ibn Sina University Hospital, Faculty of Medicine and Pharmacy, Mohammed V University, Rabat, Morocco

**Keywords:** auto-inflammatory disease, therapy, new insights, review, registries

The concept of autoinflammation was introduced in McDermott et al. (1) and refers to primary diseases of innate immunity caused by the activation of the inflammasome with the production of cytokines. The diseases are induced by inappropriate activation of antigen-independent inflammatory mechanisms. However, cells associated with adaptative immunity (e.g., T lymphocytes) may also contribute to autoinflammation (2, 3).

The demonstration of the genetic origin of these rare diseases has made it possible to better understand the immunopathogenesis of autoinflammatory diseases (AIDs).

Monogenic AIDs are caused by mutations of genes coding for proteins, which play a role in the regulation of the inflammatory response. Most AIDs have an early onset and make a clinical picture of recurrent fevers associated with inflammatory cutaneous, mucosal, serosal, and osteoarticular involvement and a long-term risk of secondary amyloidosis. These clinical abnormalities occur in the form of repeated attacks and are associated with a biological inflammatory syndrome not explained by an infectious or autoimmune cause. Apart from crises, patients are asymptomatic without systemic inflammation (4).

Familial Mediterranean fever (FMF), cryopyrin-associated periodic fever syndrome or NLRP3-associated AIDs, mevalonate-kinase deficiency, and TNFRSF1A-receptor associated periodic fever syndrome are the first four described monogenic diseases and are considered under the term periodic fevers. They correspond to a disorder of the inflammasomes and are related to IL-1 family cytokines.

Other AIDs associate systemic inflammation and skin damage such as urticaria rash with other clinical manifestations. It is the case of familial cold autoinflammatory syndrome, Muckle-Wells syndrome, and chronic infantile neurological cutaneous and articular syndrome (CINCA).

New diseases are also currently being described, due to advances in genetics analysis and may be categorized into three working groups depending on the pathogenic mechanisms involved. Thus, we distinguish the pathologies mediated by IL-1 family cytokines (e.g., the case of FMF or mevalonate-kinase deficiency), the diseases of interferon production and signaling called interferonopathies [e.g., Aicardi-Goutières syndrome and STING-associated vasculitis with onset in infancy (SAVI)], and the diseases of NFkB activation [e.g., Blau syndrome and haploinsufficiency of A20/tumor necrosis factor alpha-induced protein 3 (TNFAIP3)]. The clinical presentation of these diseases is variable, with recurrent fever and with dominant skin involvement or as an immune deficiency (2–5). The principal inflammatory mechanism linked to each disease is targeted for treatment and includes the use of biological agents that block different cytokines.

Additional disorders are also classified as autoinflammatory syndrome with or without identifiable genetic cause. However, some autoinflammatory diseases result from multiple mechanisms and do not neatly fall into the categories listed above.

For example, etiologic defects have been identified for cyclic neutropenia, pyogenic arthritis, pyoderma gangrenosum and acne (PAPA) syndrome, pyoderma gangrenosum, acne and suppurative hidradenitis (PASH) syndrome, deficiency of the IL-1 receptor antagonist (IL-1Ra) (DIRA), deficiency of the IL-36R antagonist (DITRA) and recently identified vacuoles, E1 enzyme/X-linked autoinflammatory somatic (or VEXAS) syndrome (5, 6). Those without a known cause include, for example, systemic-onset juvenile idiopathic arthritis, adult-onset Still disease, periodic fever, aphthous stomatitis, pharyngitis and adenopathy (PFAPA), non-infectious uveitis, non-infectious scleritis, undifferentiated autoinflammatory diseases, Behçet disease, and Schnitzler syndrome.

Given the rarity of systemic monogenic and multifactorial AIDs, the creation of international registries was a necessity. These registries have the advantage of including a larger number of patients and of globally sharing broad knowledge on the management of these rare diseases through the sharing of experience of clinicians and researchers. This is the case of the AutoInflammatory Disease Alliance (AIDA) network that will be presented in this issue (7).

The current Research Topic presents several registries including The Autoinflammatory Disease Alliance Registry of Monogenic Autoinflammatory Disease, the AIDA International Registry for patients with Schnitzler's syndrome, with Behçet's disease, with undifferentiated systemic autoInflammatory disease, and with Still's disease, the AIDA International registry for patients with VEXAS syndrome, and the AIDA registry for patients with axial spondyloarthritis in patients with recurrent fever attacks.

In addition, the current collection includes a prospective multicenter study from France about vasculitis and familial Mediterranean fever. The study, which includes 22 patients with both FMF and vasculitis, showed that polyarteritis nodosa (PAN) (*n* = 10) and IgA vasculitis (*n* = 8) were predominant with a high frequency of bleeding in FMF-associated PAN. The authors concluded that FMF should be investigated in case of persistent symptoms and/or inflammatory syndrome despite vasculitis treatment in Mediterranean patients.

We also invite you to read in this issue, a very interesting review by Naga et al. about the diagnosis and management of Behçet uveitis.

Contributors and editors of this Research Topic invite readers to take advantage of this collection and read up-to-date information about a variety of research areas on AIDs. Clinical advances, including advances in the treatment of AIDs, will be covered with a focus on all new insights in the field.

Disease-specific therapeutic strategies are established for some AIDs, but new therapeutic approaches are needed. An article included in this Research Topic reviewed the effectiveness and safety of JAK Inhibitors (JAKi) in AIDs. Through a systematic review of the literature in accordance with the PRISMA guidelines, the results show that JAKi can be beneficial in certain AIDs. The risk of viral infections should be considered. To accurately assess the risk-benefit ratio of JAKi for AIDs, clinical trials should be conducted.

The effectiveness of JAK inhibitors was evaluated in a Chinese study involving six patients with trisomy 8 and autoinflammatory features, with a favorable response in 4/6 patients with glucocorticoid sparing effect and good tolerance.

Additionally, a real-life study from the International AIDA Registry assessed any difference in the effectiveness of the IL-1β antagonist (canakinumab) prescribed as a first-line biologic agent between systemic and chronic-articular Still's disease. The results of the study of 26 patients showed that, when used as a first-line biotechnological agent, canakinumab has proved to be effective in controlling both clinical and laboratory manifestations regardless of the type of disease course.

In conclusion, approaches to diagnosis and therapeutic management of AIDs are rapidly developing. It is expected that this research will spark new and very interesting studies to better understand AIDs and how to manage patients.

## Author contributions

WA designed, wrote and reviewed the article, and collaborated with the writing and the revision of the manuscript.
